# Clinical outcomes with an aspheric monofocal and a new enhanced monofocal intraocular lens with modified optical profile

**DOI:** 10.1007/s00417-023-06128-8

**Published:** 2023-05-31

**Authors:** Oege Goslings, Henk Veraart, Janny van de Laar-Muskens, David P. Piñero

**Affiliations:** 1 Department of Ophthalmology, Elisabeth-TweeSteden Hospital, Hilvarenbeekseweg 60, 5022 GC Tilburg, The Netherlands; 2 Department of Optics, Pharmacology and Anatomy, University of Alicante, Crta San Vicente del Raspeig S/N 03016, San Vicente del Raspeig, Alicante, Spain

**Keywords:** Monofocal IOL, Cataract surgery, Patient-reported outcomes, Catquest-9SF, Tecnis Eyhance, Depth of focus

## Abstract

**Purpose:**

This study aimed to evaluate and compare the clinical outcomes obtained after cataract surgery with an aspheric monofocal intraocular lens (IOL) and an enhanced IOL with a modified optical profile.

**Methods:**

Randomised clinical trial enrolling 70 patients (age, 52–87 years) undergoing cataract surgery. Two groups were created according to the type of IOL implanted: Vivinex iSert from Hoya Surgical Optics (Vivinex group, 35 patients) and Tecnis Eyhance ICB00 from Johnson & Johnson Vision (Eyhance group, 35 patients). Uncorrected (UDVA) and corrected distance visual acuity (CDVA), uncorrected (UIVA) and distance-corrected intermediate visual acuity (DCIVA), refraction, and self-perceived visual function (Catquest-9SF) were evaluated during a 3-month follow-up.

**Results:**

No significant differences were found between IOL groups in UDVA and CDVA (*p* ≥ 0.093). In contrast, monocular and binocular UIVA and DCIVA were significantly better in the Eyhance group at 1 (*p* ≤ 0.015) and 3 months postoperatively (*p* ≤ 0.002). Postoperative DCIVA 20/25 or better was obtained in 71.4% and 20.0% of patients in Eyhance and Vivinex groups, respectively. Differences in postoperative Rasch calibrated Catquest scores between Eyhance and Vivinex groups did not reach statistical significance (*p* ≥ 0.102). However, significant correlations were only found between the change in UIVA and Catquest scores (0.364 ≤ *r* ≤ 0.444, *p* ≤ 0.041) in the Eyhance group.

**Conclusions:**

The modified monofocal IOL evaluated provides better intermediate visual function in comparison with a standard aspheric monofocal IOL, but the impact of this benefit on the self-perceived level of vision achieved after surgery according to the patient seemed to be limited.


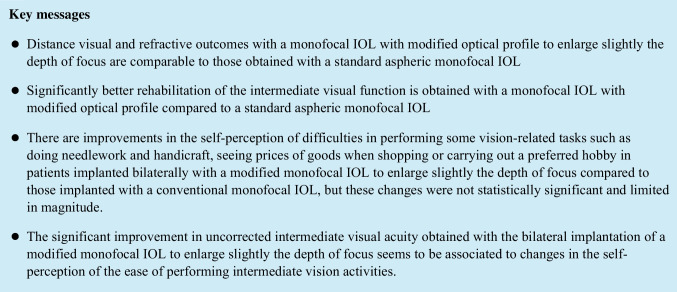


## Introduction

New designs in intraocular lens (IOL) have been developed in recent years aimed at providing patients with the highest level of spectacle independence after cataract surgery. Multifocal IOLs have demonstrated to be an effective option to restore the visual function after cataract surgery [1], being then a good option for presbyopia correction [2]. Despite the high efficacy of these implants for the restoration of the distance, intermediate and near visual function, the perception of unwanted visual phenomena, such as glare or halos, is still considered a relevant limitation [3, 4]. Indeed, this is one of the main causes of dissatisfaction after cataract surgery with multifocal IOL implantation [5], and a potential reason for IOL explantation [6]. Extended depth of focus (EDOF) IOLs were developed to overcome this limitation by inducing a continuous range of focus, but with a not completely functional near vision compared to multifocal IOLs [7]. However, although EDOF IOLs can provide good distance and intermediate visual outcomes as well as a functional near visual outcome [7–13], they are not completely free from photic phenomena [14].

Recent advances in the development of IOLs are focused on trying to provide good distance and functional intermediate visual quality, without the induction of photic phenomena. One of these new IOLs is the Tecnis Eyhance IOL from Johnson & Johnson Vision, which is a monofocal lens incorporating a spherical posterior surface and higher order aspheric anterior surface designed to create a continuous increase in power from the periphery to the centre of the lens [15, 16]. Several studies have demonstrated that this IOL provides an enhanced intermediate visual function and similar distance performance and photic phenomena when compared with standard monofocal IOLs, in most of cases from the same company [17–27]. However, differences with other types of aspheric monofocal IOLs and the impact of such differences on patient-reported outcomes (PROMs) have not been investigated in detail [17, 18]. The aim of the current study was to compare the clinical outcomes, especially intermediate visual acuity which is a critical factor for classifying IOLs as EDOF or “monofocal-EDOF” or “monofocal plus” IOLs [28], as well as PROMs between patients implanted bilaterally after cataract surgery with the Tecnis Eyhance IOL and eyes implanted with a an aspherical monofocal IOL from another different manufacturer, not investigating only differences in terms of clinical outcomes but also on how the patient perceives the improvement achieved with each IOL.

## Methods

### Patients

This study was a prospective, comparative, randomised clinical trial that was conducted in the Elisabeth-TweeSteden Hospital, Tilburg, the Netherlands. A total of 70 patients with an age ranging from 52 to 87 years old and requiring cataract surgery were recruited and included in the study consecutively. All of them were informed about the nature of the study and signed an informed consent prior to their inclusion following the tenets of the Declaration of Helsinki. Furthermore, the study protocol was revised and approved by the ethics committee of the county of Brabant (METC Brabant) (Reference number: NL70320.028.19).

Patients were eligible for inclusion in the study if they suffered from bilateral cataract, the surgery in the second eye was planned to be performed within 2 weeks after the first eye, the bilateral cataract surgery was expected to be uncomplicated, and the following conditions were present: corneal astigmatism of less than 1 D, axial length between 21 and 27 mm, and mean corneal power between 41 and 47 D. Regarding exclusion criteria, the following conditions were considered: age < 18 years; insufficient understanding of the Dutch language to comply with study procedures and/or complete patient questionnaires; inability to complete follow-up or comply with study procedures; non-routine cataract surgery (e.g. cataract surgery combined with another ocular procedure); cognitive or behavioural conditions that might interfere with surgery; conditions that increase the risk of endophthalmitis, such as current ocular, adnexal, or periocular infections (e.g. untreated blepharitis); immune-compromised (e.g. systemic corticosteroid use, leukaemia) or iodine allergy; factors that increase the risk of refractive surprise, such as abnormal axial lengths (< 21 mm or > 27 mm) or a difference between both eyes of > 1.5 mm, abnormal keratometry readings, previous refractive surgery, or myopia with posterior staphylomas; conditions that increase the risk of corneal oedema (e.g. Fuchs’ endothelial dystrophy); factors that increase the risk of complicated surgery, such as previous ocular surgery, previous perforating, or blunt eye trauma, eye, adnexal, or anatomical abnormalities (including pseudoexfoliation syndrome); lens luxation or iridodonesis, or cataract nigrans-posterior polar cataract, sight-threatening comorbidity, glaucoma, or intraocular pressure of > 24 mm Hg, uveitis, diabetes mellitus with diabetic retinopathy and macular oedema; and any macular disease.

Patients who were willing to participate and met the inclusion and exclusion criteria were randomised to either the implantation of the Vivinex iSert© (Hoya Surgical Optics, Chromos, Singapore) or Tecnis Eyhance ICB00© (Johnson & Johnson Vision, Jacksonville, FL, USA) IOL. Each patient received a randomization number from a computerised random number generator. In case a patient did not want to be randomised, he or she was not included in this study. The follow-up duration per patient was up to 3 months after surgery.

### Intraocular lenses

The Tecnis Eyhance IOL is an acrylic hydrophobic one-piece, foldable IOL with a total diameter of 13.0 mm and an optic diameter of 6.0 mm. It has a spherical posterior surface and a modified aspheric anterior surface which consists of a continuous increase in power from the periphery to the centre of the lens to maintain distance image quality comparable to aspheric monofocal IOLs [29]. In an optical bench study, it was demonstrated that this anterior surface is modified with 0.50 D of additional power in its central 2-mm zone, which induces an increased image quality in the intermediate range [15], but this information is not confirmed by the manufacturer. A dioptric range from + 5.0 D to + 34.0 D, in 0.5 diopter increments is available. The optical A-constant for Tecnis Eyhance is 119.3 [29].

The Vivinex iSert IOL is an acrylic single-piece aspheric IOL with an optic size of 6.0 mm and an aspheric balance curve design, which was intended to reduce coma aberrations in addition to spherical aberration. Specifically, this IOL provides − 0.18 μm (6.0 mm pupil) of spherical aberration correction [30] in an attempt to compensate for the normal level of corneal spherical aberration (range, 0.058 to 0.545 µm for 6-mm pupil) [31]. A dioptric range from + 6.0 D to + 30.0 D, in 0.5 diopter increments, is available. The optical A-constant for Vivinex iSert is 118.9.

### Clinical protocol

Patients participating in this study performed the following visits:

- Preoperative: Patients were checked for inclusion and exclusion criteria. In this visit, a standard preoperative assessment was performed including medical history, measurement of monocular and binocular uncorrected (UDVA) and corrected distance visual acuity (CDVA) using the ETDRS test, measurement of monocular and binocular uncorrected (UIVA) and distance-corrected intermediate visual acuity (DCIVA) using the ETDRS test at 66 cm, subjective refraction, slit lamp biomicroscopy, Goldmann tonometry, and fundus evaluation. In addition, participants filled out the Catquest-9 SF questionnaire, which is a validated tool to evaluate the visual function outcome reported by the patient [32].
- One week after first eye surgery and 4/6 weeks after second eye surgery: standard postoperative assessment that is required after cataract surgery, including postoperative evaluation of UDVA, CDVA, UIVA, and DCIVA, subjective refraction, Goldmann tonometry, and slit lamp biomicroscopy.
- 3 months after surgery: complete ophthalmological examination including the same tests as in the preoperative assessment.

The Catquest questionnaire contains 9 questions within 4 areas asking about the perceived difficulty in performing some daily-life activities and global satisfaction with vision [32]. Each question about difficulties in doing tasks has the following answer options and scores associated: 4 = very great difficulty, 3 = great difficulty, 2 = some difficulty, and 1 = no difficulty. For satisfaction with vision, the 4 response options are as follows: 4 = very dissatisfied, 3 = rather dissatisfied, 2 = fairly satisfied, and 1 = very satisfied [32]. These scores were transformed into Rasch calibrated scores using a 4-Andrich rating scale design, as previously described by the authors who developed the questionnaire [32]. Rasch calibrated scores consider that the items are of varying difficulty and that the distance between the response options is not equally big, allowing their treatment with parametric statistics. Regarding the interpretation of the scale, higher values are associated with higher levels of difficulty in doing vision-related tasks and lower levels of patient satisfaction [32].

### Surgical procedure

The intervention consisted of cataract surgery performed by an experienced cataract surgeon using a phacoemulsification technique. All the procedures were executed by the same surgeon (HV). In each patient, the operations of each eye were done on separate days (delayed sequential bilateral cataract surgery). A standard technique of sutureless microincision phacoemulsification was used. Surgeries were initiated after instilling anaesthesia and mydriatic drops by performing a corneal incision at the temporal area. The procedure was followed with the creation of the capsulorhexis and the performance of the phacoemulsification. After this, the IOL was inserted into the capsular bag through the main incision using the injector developed by the manufacturer for this purpose.

A postoperative topical therapy based on a combination of topical antibiotic and steroid was prescribed to be applied four times daily for one week.

### Statistical analysis

The sample of eyes included in this study had to be of adequate size, relative to the goals of the study. For the determination of the required sample size for the study described in this protocol, data from a preliminary study (6 months of follow-up) conducted at 9 sites in Europe (142 patients) aimed at evaluating and comparing the visual outcomes in eyes implanted with the modified aspheric monofocal IOL Tecnis Eyhance ICB00 and with the monofocal IOL Tecnis ZCB00 were considered. The Dupont and Plummer approach was used for the sample size calculation [33]. For an unpaired *t*-test; a statistical power of 90%; expected mean postoperative values of DCIVA of 0.09 and 0.20 logMAR in the ICB00 and ZCB00 groups, respectively; a within group standard deviation of 0.13 logMAR; and alpha error of 0.05, a sample size of 30 per group was necessary. Considering a potential patient drop-out rate of 15%, the sample size per group required was 35 eyes. As both eyes of the same individual were included, a total of 35 patients (70 eyes) was necessary. DCIVA was considered as the main outcome measure for the basis of this calculation, as this parameter is crucial for characterizing and differentiating EDOF, mono-EDOF, and standard monofocal IOLs [28]. According to the American Academy of Ophthalmology Task Force Consensus Statement for Extended Depth of Focus Intraocular Lenses, an IOL cannot be considered as EDOF and could be considered as a “monocular EDOF” or “monocular plus” IOL if a minimum of 100 patients are implanted with the IOL and compared with a monofocal IOL, not obtaining at least 50% of eyes with monocular DCIVA of 0.2 logMAR (20/32) or better at 66 cm [28].

Statistical analyses were performed with a commercially available software package (SPSS for Mac, Version 20.0; IBM Corporation, Armonk, NY, USA). Normality of data samples was evaluated by means of the Kolmogorov–Smirnov test. When parametric analysis was possible, the Student *t* test for paired data was used for comparisons between consecutive visits in each IOL group, whereas the Wilcoxon ranked sum test was applied to assess the significance of such differences when parametric analysis was not possible. Likewise, when parametric analysis was possible, differences between the two IOL groups were evaluated using the unpaired Student’ *t* test. Otherwise, if parametric analysis was not possible, differences between IOL groups were assessed with the Mann–Whitney test. Pearson or Spearman correlation coefficients (with or without the assumption of normality of data samples, respectively) were used to analyse the strength of the relationship between different variables. Differences were considered as statistically significant when the associated *p*-value was < 0.05.

Concerning refraction notation, the power vector method described by Thibos and Horner was used for converting the preoperative and postoperative spherocylindrical refractions [34]. In this method, any spherocylindrical refractive error can be expressed by a vector in a 3-dimensional dioptric space (*M* or SE, *J*_0_, *J*_45_), being the length of this vector a measure of the overall blurring strength (*B*) of a spherocylindrical refractive error. *M* is the spherical equivalent refraction error, and *J*_0_ and *J*_45_ are the 2 Jackson crossed cylinders equivalent to the conventional cylinder. Specifically, manifest refractions in conventional script notation (*S* [sphere], *C* [cylinder], *φ* [axis]) were converted to power vector coordinates and overall blurring strength using the following formulas: *M* or SE = *S* + *C*/2; *J*_0_ = (− C/2) cos(2*φ*); *J*_45_ = (− *C*/2) sin(2*φ*); *B* = (*M*^2^ + *J*_0_^2^ + *J*_45_^2^)^1/2^.

## Results

A total of 140 eyes of 70 patients with a mean age of 71.8 years (SD: 7.2, median: 72.0, range: 52 to 87 years) were enrolled. The sample comprised 35 males (50.0%) and 35 females (50.0%). Two groups were created depending on the IOL implanted: Eyhance group, including 70 eyes of 35 patients implanted with the Tecnis Eyhance ICB00 IOL, and Vivinex group, including 70 eyes of 35 patients implanted with the Vivinex iSert IOL. Table 1 summarises the preoperative data obtained in both groups. As shown, no statistically significant parameters between IOL groups were found in any of the clinical parameters evaluated (*p* ≥ 0 0.177). Tables 2 and 3 provide the visual and refractive data obtained at 1 month and 3 months after surgery, respectively.

**Table 1:** Summary of Preoperative clinical data in the two groups defined in the current study. The corresponding *p*-values for the comparison between groups are shown for each parameter evaluated

Mean (SD) Median (range)	Eyhance group	Vivinex group	*p*-value
Age (years)	72.4 (6.7) 74.0 (55.0 to 81.0)	71.3 (7.6) 71.0 (52.0 to 87.0)	0.498
Gender (male/female)	18/17	17/18	0.811
Monocular LogMAR UDVA	0.56 (0.35) 0.45 (0.04 to 1.38)	0.60 (0.39) 0.50 (0.06 to 1.56)	0.334
Binocular LogMAR UDVA	0.32 (0.29) 0.20 (0.04 to 1.10)	0.36 (0.25) 0.32 (0.00 to 1.02)	0.267
Monocular LogMAR CDVA	0.21 (0.18) 0.19 (− 0.16 to 1.04)	0.20 (0.16) 0.18 (− 0.10 to 0.64)	0.577
Binocular LogMAR CDVA	0.08 (0.11) 0.06 (− 0.14 to 0.36)	0.08 (0.10) 0.10 (− 0.12 to 0.32)	0.995
Monocular LogMAR UIVA	0.60 (0.28) 0.53 (0.08 to 1.20)	0.57 (0.30) 0.56 (0.00 to 1.20)	0.423
Binocular LogMAR UIVA	0.47 (0.27) 0.44 (− 0.02 to 1.16)	0.43 (0.25) 0.38 (0.08 to 1.10)	0.365
Monocular LogMAR DCIVA	0.43 (0.15) 0.42 (0.18 to 0.98)	0.40 (0.15) 0.38 (0.14 to 0.82)	0.177
Binocular LogMAR DCIVA	0.34 (0.12) 0.34 (0.12 to 0.60)	0.30 (0.11) 0.30 (0.06 to 0.58)	0.193
Sphere (D)	0.55 (2.53) 0.88 (− 7.75 to 5.25)	0.70 (2.44) 1.00 (− 7.50 to 6.50)	0.988
Cylinder (D)	− 0.90 (0.65) − 0.75 (− 3.50 to 0.00)	− 0.74 (0.54) − 0.75 (− 2.00 to 0.00)	0.246
Spherical equivalent (D)	0.10 (2.58) 0.50 (− 8.50 to 5.00)	0.33 (2.44) 0.56 (− 7.50 to 5.75)	0.803
*J*_0_ (D)	− 0.19 (0.40) − 0.15 (− 1.12 to 0.98)	− 0.24 (0.33) − 0.24 (− 1.00 to 0.47)	0.524
*J*_45_ (D)	− 0.01 (0.34) 0.00 (− 1.34 to 0.86)	− 0.07 (0.21) 0.00 (− 0.62 to 0.60)	0.223
*B* (D)	1.99 (1.72) 1.63 (0.18 to 8.56)	1.97 (1.53) 1.54 (0.00 to 7.50)	0.868

*SD* standard deviation, *D* diopters, *UDVA* uncorrected distance visual acuity, *UIVA* uncorrected intermediate visual acuity, *CDVA* corrected distance visual acuity, *DCIVA* distance-corrected intermediate visual acuity, *J*_0_ and *J*_45_ power vectors, *B* overall blur strength

**Table 2:** Summary of 1-month postoperative clinical data in the two groups defined in the current study. The corresponding *p*-values for the comparison between groups are shown for each parameter evaluated

Mean (SD) Median (range)	Eyhance group	Vivinex group	*p*-value
Monocular LogMAR UDVA	0.12 (0.14) 0.10 (− 0.12 to 0.70)	0.11 (0.14) 0.10 (− 0.12 to 0.48)	0.921
Binocular LogMAR UDVA	0.03 (0.10) − 0.01 (− 0.10 to 0.30)	0.05 (0.17) 0.04 (− 0.14 to 0.86)	0.824
Monocular LogMAR CDVA	0.03 (0.11) 0.00 (− 0.16 to 0.50)	− 0.01 (0.08) 0.00 (− 0.14 to 0.26)	0.093
Binocular LogMAR CDVA	− 0.05 (0.06) − 0.04 (− 0.18 to 0.08)	− 0.06 (0.07) − 0.06 (− 0.20 to 0.06)	0.887
Monocular LogMAR UIVA	0.26 (0.15) 0.24 (0.02 to 0.80)	0.30 (0.11) 0.31 (0.04 to 0.60)	0.015
Binocular LogMAR UIVA	0.13 (0.11) 0.10 (− 0.04 to 0.40)	0.20 (0.11) 0.20 (0.00 to 0.60)	0.001
Monocular LogMAR DCIVA	0.27 (0.14) 0.26 (0.00 to 0.80)	0.37 (0.12) 0.37 (0.10 to 0.74)	< 0.001
Binocular LogMAR DCIVA	0.16 (0.13) 0.16 (− 0.06 to 0.42)	0.28 (0.12) 0.28 (0.00 to 0.62)	< 0.001
Sphere (D)	0.31 (0.44) 0.25 (− 0.50 to 1.50)	0.13 (0.43) 0.13 (− 0.75 to 1.00)	0.031
Cylinder (D)	− 0.66 (0.51) − 0.75 (− 1.50 to 0.00)	− 0.65 (0.49) − 0.50 (− 2.25 to 0.00)	0.656
Spherical equivalent (D)	− 0.02 (0.34) 0.00 (− 0.62 to 0.75)	− 0.19 (0.39) − 0.19 (− 1.00 to 0.50)	0.023
*J*_0_ (D)	− 0.13 (0.32) − 0.10 (− 0.75 to 0.59)	− 0.15 (0.32) − 0.12 (− 0.97 to 0.75)	0.739
*J*_45_ (D)	0.04 (0.24) 0.00 (− 0.57 to 0.74)	0.05 (0.20) 0.00 (− 0.35 to 0.64)	0.787
*B* (D)	0.47 (0.25) 0.50 (0.00 to 1.06)	0.53 (0.27) 0.53 (0.00 to 1.03)	0.484

*SD* standard deviation, *D* diopters, *UDVA* uncorrected distance visual acuity, *UIVA* uncorrected intermediate visual acuity, *CDVA* corrected distance visual acuity, *DCIVA* distance-corrected intermediate visual acuity, *J*_0_ and *J*_45_ power vectors, *B* overall blur strength

**Table 3:** Summary of 3-month postoperative clinical data in the two groups defined in the current study. The corresponding *p*-values for the comparison between groups are shown for each parameter evaluated

Mean (SD) Median (range)	Eyhance group	Vivinex group	*p*-value
Monocular LogMAR UDVA	0.09 (0.13) 0.07 (− 0.14 to 0.50)	0.10 (0.13) 0.09 (− 0.08 to 0.40)	0.889
Binocular LogMAR UDVA	0.01 (0.11) 0.02 (− 0.18 to 0.30)	0.00 (0.08) − 0.02 (− 0.16 to 0.20)	0.685
Monocular LogMAR CDVA	0.00 (0.10) 0.00 (− 0.18 to 0.30)	− 0.02 (0.11) − 0.04 (− 0.24 to 0.30)	0.167
Binocular LogMAR CDVA	− 0.05 (0.09) − 0.05 (− 0.18 to 0.20)	− 0.08 (0.07) − 0.08 (− 0.24 to 0.10)	0.752
Monocular LogMAR UIVA	0.24 (0.15) 0.22 (− 0.06 to 0.66)	0.32 (0.13) 0.30 (0.06 to 0.64)	0.001
Binocular LogMAR UIVA	0.12 (0.11) 0.12 (− 0.10 to 0.44)	0.22 (0.12) 0.19 (0.00 to 0.48)	0.001
Monocular LogMAR DCIVA	0.23 (0.13) 0.22 (− 0.14 to 0.48)	0.33 (0.12) 0.34 (0.10 to 0.60)	0.002
Binocular LogMAR DCIVA	0.12 (0.11) 0.12 (− 0.16 to 0.40)	0.24 (0.10) 0.24 (0.10 to 0.42)	< 0.001
Sphere (D)	0.44 (0.47) 0.50 (− 0.50 to 1.50)	0.17 (0.45) 0.13 (− 0.75 to 1.25)	0.002
Cylinder (D)	− 0.75 (0.48) − 0.75 (− 2.00 to 0.00)	− 0.58 (0.42) − 0.50 (− 1.75 to 0.00)	0.019
Spherical equivalent (D)	0.06 (0.39) 0.00 (− 0.88 to 1.12)	− 0.12 (0.37) − 0.13 (− 0.88 to 0.75)	0.006
J_0_ (D)	− 0.13 (0.34) − 0.12 (− 0.99 to 0.62)	− 0.17 (0.27) − 0.14 (− 0.86 to 0.37)	0.718
J_45_ (D)	− 0.02 (0.26) 0.00 (− 0.62 to 0.59)	0.00 (0.16) 0.00 (− 0.38 to 0.35)	0.732
B (D)	0.51 (0.29) 0.53 (0.00 to 1.13)	0.48 (0.22) 0.45 (0.00 to 0.95)	0.251

*SD* standard deviation, *D* diopters, *UDVA* uncorrected distance visual acuity, *UIVA* uncorrected intermediate visual acuity, *CDVA* corrected distance visual acuity, *DCIVA* distance-corrected intermediate visual acuity, *J*_0_ and *J*_45_ power vectors, *B* overall blur strength

### Refractive outcomes

**Fig. 1:**
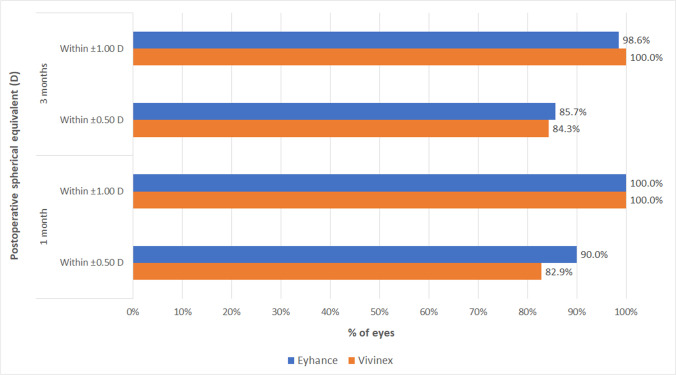
Distribution of postoperative spherical equivalent in the two groups of eyes evaluated in the current study

Significant differences were found between IOL groups in postoperative sphere and spherical equivalent (*p* ≤ 0.031), with a trend to a less myopic residual refractive error in the Eyhance group in the two postoperative visits (Tables 2 and 3). However, no significant differences between IOL groups were found postoperatively in power vector components of manifest cylinder (*p* ≥ 0.718). The difference in the magnitude of manifest cylinder between IOL groups only reached statistical significance at 3 months after surgery (*p* = 0 0.019). At the end of the follow-up, a total of 85.7% and 84.3% of eyes achieved a postoperative spherical equivalent within ± 0.50 D in Eyhance and Vivinex groups, respectively (Fig. 1), respectively.

### Visual outcomes

**Fig. 2:**
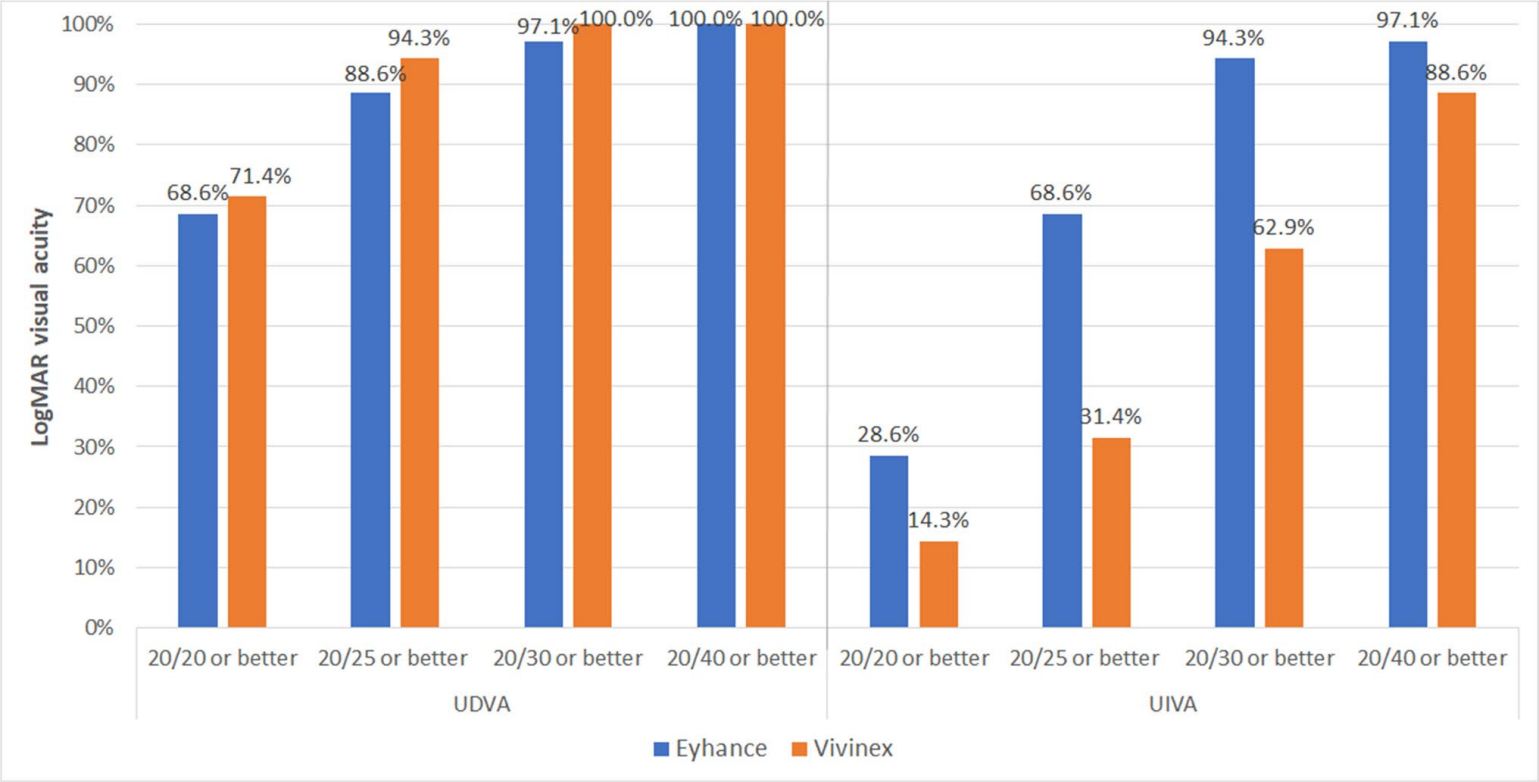
Distribution of 3-month postoperative binocular uncorrected distance (UDVA) and intermediate visual acuity (UIVA) in the two groups of patients evaluated in the current study

**Fig. 3:**
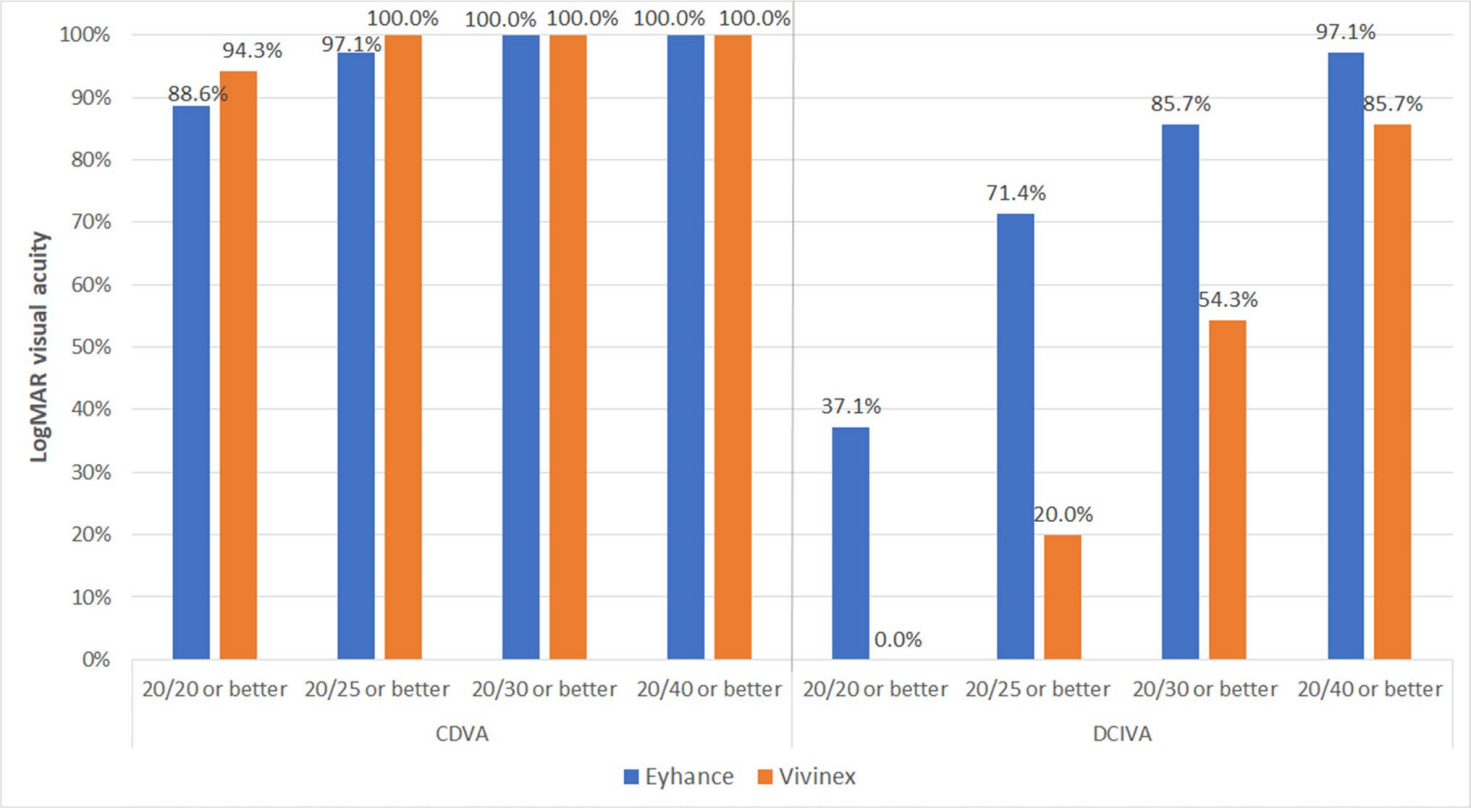
Distribution of 3-month postoperative binocular corrected distance (CDVA) and distance-corrected intermediate visual acuity (DCIVA) in the two groups of patients evaluated in the current study

No significant differences were found between IOL groups in monocular and binocular UDVA and CDVA (*p* ≥ 0.093). In contrast, monocular and binocular UIVA and DCIVA values were significantly better in the Eyhance group at 1 (*p* ≤ 0.015) and 3 months after surgery (*p* ≤ 0.002). At the end of the follow-up, a total of 88.6% and 94.3% of patients achieved a binocular UDVA of 20/25 or better in the Eyhance and Vivinex groups, respectively, whereas a total of 68.6% and 31.4% achieved a binocular UIVA of 20/25 or better (Fig. 2). Regarding binocular CDVA, a total of 97.1% and 100% of patients achieved values of 20/25 or better at 3 months after surgery in Eyhance and Vivinex groups, respectively (Fig. 3). Likewise, 3-month postoperative binocular DCIVA was 20/25 or better in 71.4% and 20.0% of patients in Eyhance and Vivinex groups, respectively (Fig. 3).

### Patient-reported outcomes

Table 4 shows the preoperative and postoperative Rasch calibrated scoring obtained with the Catquest-9SF questionnaire in the two groups defined in the current study. As shown, only statistically significant differences were found preoperatively between IOL groups for the Rasch calibrated scores corresponding to the Item B (*p* = 0.009). At 3 months after surgery, Rasch calibrated scores for each item and the total scores were somewhat better in the Eyhance group compared to Vivinex group, but the differences did not reach statistical significance (*p* ≥ 0.104).

**Table 4:** Summary of preoperative and postoperative Rasch calibrated scoring obtained with the Catquest-9SF questionnaire in the two groups defined in the current study. The corresponding *p*-values for the comparison between groups are shown for each parameter evaluated

Mean (SD) Median (range)	Preoperative			Postoperative		
	Eyhance group	Vivinex group	*p*-value	Eyhance group	Vivinex group	*p*-value
Item A: Do you experience that your present vision gives you difficulties in any way in your daily life?	− 0.96 (1.69) − 1.26 (− 3.98 to 3.22)	− 0.94 (1.49) − 1.26 (− 3.98 to 3.22)	0.879	− 3.48 (1.25) − 3.98 (− 3.98 to 1.21)	− 3.18 (1.26) − 3.98 (− 3.98 to − 1.26)	0.217
Item B: Are you satisfied or dissatisfied with your present vision?	1.85 (1.48) 2.67 (0.20 to 4.67)	2.73 (1.19) 2.67 (0.20 to 4.67)	0.009	− 1.45 (1.76) − 2.53 (− 2.53 to 2.67)	− 1.42 (1.62) − 2.53 (− 2.53 to 2.67)	0.828
Item C1: Do you have difficulty with reading text in the newspaper because of your vision?	− 2.02 (2.33) − 1.46 (− 4.18 to 3.02)	− 1.81 (2.48) − 1.46 (− 4.18 to 3.02)	0.749	− 3.34 (1.43) − 4.18 (− 4.18 to 3.02)	− 3.32 (1.68) − 4.18 (− 4.18 to 1.01)	0.795
Item C2: Do you have difficulty with recognizing faces of people you meet because of your vision?	− 1.98 (2.43) − 3.63 (− 3.63 to 3.57)	− 1.87 (2.20) − 3.63 (− 3.63 to 3.57)	0.712	− 3.38 (0.81) − 3.63 (− 3.63 to − 0.91)	− 3.39 (0.78) − 3.63 (− 3.63 to − 0.91)	0.938
Item C3: Do you have difficulty with seeing prices of goods when shopping because of your vision?	− 2.89 (2.00) − 4.44 (− 4.44 to 2.76)	− 2.31 (2.41) − 1.72 (− 4.44 to 2.76)	0.267	− 3.76 (1.20) − 4.44 (− 4.44 to − 1.72)	− 3.36 (1.94) − 4.44 (− 4.44 to 0.75)	0.638
Item C4: Do you have difficulty with seeing to walk on uneven ground because of your vision?	− 2.53 (1.80) − 3.77 (− 3.77 to 3.42)	− 2.45 (1.96) − 3.77 (− 3.77 to 1.42)	0.921	− 3.60 (0.67) − 3.77 (− 3.77 to − 1.05)	− 3.53 (0.78) − 3.77 (− 3.77 to − 1.05)	0.695
Item C5: Do you have difficulty with seeing to do needlework and handicraft because of your vision?	− 1.80 (2.44) − 3.37 (− 3.37 to 3.83)	− 1.36 (2.09) − 0.65 (− 3.37 to 3.83)	0.155	− 2.82 (1.30) − 3.37 (− 3.37 to 1.82)	− 2.26 (1.62) − 3.37 (− 3.37 to 1.82)	0.117
Item C6: Do you have difficulty with reading text on television because of your vision?	− 2.47 (2.41) − 2.95 (− 4.59 to 1.98)	− 1.75 (2.98) − 1.31 (− 4.59 to 4.25)	0.286	− 4.28 (0.97) − 4.59 (− 4.59 to − 1.31)	− 4.20 (1.07) − 4.59 (− 4.59 to − 1.31)	0.755
Item C7: Do you have difficulty with seeing to carry out a preferred hobby because of your vision?	− 3.68 (2.05) − 4.95 (− 4.95 to 2.98)	− 3.35 (2.33) − 4.95 (− 4.95 to 2.98)	0.733	− 4.71 (1.02) − 4.95 (− 4.95 to 0.34)	− 4.36 (1.26) − 4.95 (− 4.95 to 0.34)	0.104
Overall score	− 1.81 (1.45) − 2.09 (− 3.33 to 3.27)	− 1.38 (1.59) − 1.88 (− 3.33 to 3.30)	0.213	− 3.43 (0.63) − 3.64 (− 3.94 to − 1.24)	− 3.23 (0.73) − 3.35 (− 3.94 to − 1.33)	0.324

*SD* standard deviation, *D* diopters, *UDVA* uncorrected distance visual acuity, *UIVA* uncorrected intermediate visual acuity, *CDVA* corrected distance visual acuity, *DCIVA* distance-corrected intermediate visual acuity, *J*_0_ and *J*_45_ power vectors, *B* overall blur strength

### Correlation between visual changes and PROMs

In the Eyhance group, a significant correlation of the change in the total Rasch calibrated score with the change in UIVA (*r* = 0 0.364, *p* = 0.041) was found. In contrast, in the Vivinex group, this correlation was extremely poor and not statistically significant (*r* = 0.131, *p* = 0 0.459). Furthermore, in the Eyhance group, significant correlations of the change in UDVA with the change in Rasch calibrated score for item C4 (*r* = 0.365, *p* = 0.040) was found as well as significant correlations of the change in UIVA with the changes in Rasch calibrated scores for items C2 (*r* = 0.379, *p* = 0.032) and C3 (*r* = 0.444, *p* = 0.012). However, in the Vivinex group, changes in UDVA and UIVA did not correlate significantly with changes in Rasch calibrated scores of Catquest-9SF.

## Discussion

In the current sample, an evaluation of the clinical and patient-reported outcomes with a modified monofocal IOL designed to induce some level of extension of the depth of focus has been performed, comparing such results with those obtained with an aspheric monofocal IOL from another manufacturer. To this date, this new modified monofocal IOL has been compared with the standard monofocal IOL from the same company [17, 19–21, 23–27] as well as with the hybrid EDOF IOL also from the same company (Tecnis Symfony) [19]. All these studies confirmed an enhanced intermediate visual function with the Eyhance IOL compared to the monofocal IOL. In the current study, a comparison of the modified monofocal IOL was done with an aspheric IOL from another company inducing some level of negative spherical aberration in an attempt to compensate for the spherical aberration of the cornea. Thus, it can be checked that the modified monofocal IOL is not based on a simple induction of negative spherical aberration to enlarge the depth of focus. Indeed, the modified monofocal IOL induces the same level of primary spherical aberration as the monofocal IOL of the same company for pupils larger than 3.5 mm [15]. The modified monofocal IOL has a modification of the central power profile that improves intermediate vision while maintaining comparable distance image quality and keeping the same photic phenomena profile as a standard aspheric monofocal IOL [15].

The refractive predictability obtained with both IOLs was good, with 85.7% and 84.3% of eyes achieving a 3-month postoperative spherical equivalent within ± 0.50 D with the Eyhance and Vivinex IOLs, respectively. However, the difference in postoperative sphere and SE between IOLs did reach statistical significance, although it was small in magnitude. Specifically, a trend to more myopic residual outcome with the aspheric IOL was found, which may be in relation with several factors, including the need for a refinement of the constants of the IOL required for its power calculations. It should be considered that this trend to postoperative residual myopic error in the Vivinex group might have biased the visual outcomes, leading to an enhanced intermediate visual function with this IOL and worse UDVA and UIVA. However, no significant differences between Eyhance and Vivinex groups were found in UDVA and better UIVA was found with the Eyhance IOL, revealing a minimal impact of this residual refraction on this variable. Possibly, the induction of some level of myopic residual refraction in the non-dominant eye with both IOLs would have led to better intermediate and near visual outcomes. Likewise, future studies should be performed to confirm if the bilateral implantation of the modified monofocal IOL and the implantation of the aspheric monofocal IOL with some level of micromonovision would provide similar results.

As mentioned, the distance visual outcomes were similar with both types of IOL, with mean binocular UDVA values at 3 months postoperatively of 0.03 ± 0.10 and 0.05 ± 0.17 logMAR for the Eyhance and Vivinex IOLs, respectively. Likewise, despite of the central power profile of the modified monofocal IOL to promote the extension of the depth of focus, this fact was not associated to a decrease in CDVA. Indeed, no significant differences were found in terms of postoperative monocular and binocular CDVA between the two monofocal IOLs compared. Similarly, other authors did not find either significant differences in distance visual acuity outcomes between eyes implanted with the Eyhance IOL and those implanted with different models of standard monofocal IOLs [17, 20–23, 25–27]. Cinar et al. [22] conducted a comparative study of the Eyhance IOL with the monofocal IOL SN60WF IQ AcrySof from Alcon, obtaining mean postoperative UDVA values of 0.05 ± 0.13 and 0.05 ± 0.15 logMAR, respectively. In another study conducted by Lopes et al. [17], mean average binocular UDVA of 20/22 was found in a group of patients implanted with the Eyhance IOL as well as in another group implanted with the conventional monofocal IOL from the same manufacturer (Tecnis PCB00 from Johnson & Johnson Vision). Therefore, the advanced monofocal IOL with a modified optical profile evaluated in the current sample has the same behaviour in terms of distance visual correction than a conventional monofocal IOL.

As in previous studies [17, 18, 20–23, 25–27], differences between Eyhance and conventional monofocal IOL are present when comparing UIVA and DCIVA. Specifically, in the current series, significantly better monocular and binocular UIVA and DCIVA were found during the whole postoperative follow-up in the Eyhance group compared to the Vivinex group. Indeed, the DCIVA measured binocularly at 3 months after surgery was 20/25 or better in 71.4% of patients in Eyhance group, whereas this percentage was only 20.0% in the aspheric conventional monofocal IOL group. Auffarth et al. [21] found in a European multicentre study that the Eyhance IOL significantly improved mean monocular and binocular DCIVA and UIVA by at least one logMAR line in comparison with a standard monofocal IOL (Tecnis ZCB00, Johnson & Johnson Vision). Similarly, Cinar et al. [22] reported mean values of postoperative DCIVA of 0.28 ± 0.12 and 0.38 ± 0.13 logMAR with Eyhance and standard monofocal IOLs, respectively. Likewise, some comparative studies have even reported similar results in terms of intermediate visual acuity when comparing the Eyhance IOL with EDOF IOLs [35, 36], although better near visual performance was achieved with EDOF lenses.

Besides conventional clinical outcomes, PROMs have been measured using the validated questionnaire Catquest-9SF [32]. In both groups, a significant improvement was found in the Rasch calibrated Catquest scores, which is consistent with the improvement found by previous authors in such scores after cataract surgery [18, 37, 38]. It should be considered that the replacement of the opacified crystalline lens by a transparent IOL must be necessarily associated to a self-perception of visual improvement. Lopes et al. [17] compared the results of the Catquest questionnaire in patients implanted with the Eyhance IOL and a standard monofocal IOL (Tecnis PCB00, Johnson & Johnson Vision) and found less difficulty in activities requiring intermediate vision reported by patients implanted with the Eyhance IOL. However, these authors did not analyse the Rasch calibrated scores of the questionnaire and results should be interpreted with caution. In our sample, at 3 months after surgery, Rasch calibrated scores for each item of the questionnaire and the total scores did not differ significantly in the Eyhance group compared to Vivinex group. It should be considered that the patient is asked with the Catquest how is the performance of several near and intermediate vision-related activities with correction. If a patient implanted with a monofocal IOL uses progressive addition lenses postoperatively, the questionnaire will not really measure the PROMs of the result of the surgical procedure as they would be without such spectacle correction. This might be a limiting factor to measure the satisfaction of the patients with the implant. In any case, in our sample, the change in UIVA in the Eyhance group was significantly correlated with the improvement in the total Rasch calibrated score as well as with the improvement of the Rasch calibrated scores for the items C2 (difficulty with recognizing faces of people you meet) and C3 (difficulty with seeing prices of goods when shopping), although these correlations were weak. These significant correlations were not found in the Vivinex group. This suggests that the increase in UIVA after cataract surgery with the implantation of the Eyhance IOL may be associated with an enhanced self-perceived visual function.

This study has some limitations that must be acknowledged. First, visual quality was not evaluated and compared between groups, including tests such as contrast sensitivity, photic phenomena questionnaires, or aberrometry. However, it should be considered that these aspects have been investigated previously in other comparative studies [17, 18, 20, 24]. Future studies should investigate further the relationship between visual quality measures and PROMs in eyes implanted with the Eyhance IOL. Another limitation is the sample size which was calculated considering as main outcome measure DCIVA, but possibly a somewhat higher sample would have been needed to detect significant differences between Eyhance and Vivinex groups in terms of PROMs, as these variables show a higher level of variability. Finally, a longer follow-up would be needed to confirm if this benefit of the Eyhance IOL over a conventional monofocal IOL is maintained over time.

In conclusion, the modified monofocal IOL to slightly extend the depth of focus, Tecnis Eyhance, provides a better intermediate visual function in comparison with a standard aspheric monofocal IOL, but the impact of this benefit in the self-perceived level of vision achieved after surgery according to the patient seems to be limited. No differences between modified and standard monofocal IOLs are present in terms of the distance visual rehabilitation provided.
